# Antibody Responses in COVID-19: A Review

**DOI:** 10.3389/fimmu.2021.633184

**Published:** 2021-04-15

**Authors:** Mateo Chvatal-Medina, Yorjagis Mendez-Cortina, Pablo J. Patiño, Paula A. Velilla, Maria T. Rugeles

**Affiliations:** ^1^ Grupo Inmunovirología, Facultad de Medicina, Universidad de Antioquia, Medellín, Colombia; ^2^ Grupo Inmunodeficiencias Primarias, Facultad de Medicina, Universidad de Antioquia, Medellín, Colombia

**Keywords:** therapeutics, antibodies, SARS-CoV-2, COVID-19, seroprevalence, kinetics, neutralization

## Abstract

The severe acute respiratory syndrome coronavirus 2 (SARS-CoV-2) continues to spread worldwide as a severe pandemic. Although its seroprevalence is highly variable among territories, it has been reported at around 10%, but higher in health workers. Evidence regarding cross-neutralizing response between SARS-CoV and SARS-CoV-2 is still controversial. However, other previous coronaviruses may interfere with SARS-CoV-2 infection, since they are phylogenetically related and share the same target receptor. Further, the seroconversion of IgM and IgG occurs at around 12 days post onset of symptoms and most patients have neutralizing titers on days 14-20, with great titer variability. Neutralizing antibodies correlate positively with age, male sex, and severity of the disease. Moreover, the use of convalescent plasma has shown controversial results in terms of safety and efficacy, and due to the variable immune response among individuals, measuring antibody titers before transfusion is mostly required. Similarly, cellular immunity seems to be crucial in the resolution of the infection, as SARS-CoV-2-specific CD4+ and CD8+ T cells circulate to some extent in recovered patients. Of note, the duration of the antibody response has not been well established yet.

## Introduction

Time and time again, emerging and recurring pathogens have posed a threat to humanity and materialized as global challenges to public health (1). Most often, these microorganisms are effectively contained, and their emergence does not translate into a widespread disease with high morbidity or mortality rates. Nonetheless, some few noteworthy exceptions have escaped this rule, because of their own pathophysiological nature or due to insufficient efforts at containing them. The novel coronavirus known as severe acute respiratory syndrome coronavirus 2 (SARS-CoV-2) falls into this narrow group of exceptions, as it continues to spread worldwide as a severe pandemic, with varied and often misleading estimates of its true impact (2, 3). Hence, the menace that the coronavirus disease 2019 (COVID-19) represents for global health must be met with a thorough understanding of the nature of this highly pathogenic virus, so as to focus on effective strategies that lead to its control and mitigation.

This viral pathogen has been confirmed, based on phylogenetic evidence, to be in close relationship with other highly pathogenic coronaviruses such as Middle East respiratory syndrome coronavirus (MERS-CoV) and severe acute respiratory syndrome coronavirus (SARS-CoV), with which it shares common biological features, routes of transmission and the common receptor angiotensin converting enzyme 2 (ACE-2) to infect susceptible cells (4–7). The clinical course of SARS-CoV2 infection is usually asymptomatic or with mild symptoms, including fever, cough and shortness of breath; although it can course in extreme cases with respiratory failure, requiring mechanical ventilation. Moreover, this coronavirus can also lead to several extrapulmonary manifestations, such as thromboembolic complications, cardiac lesions, acute coronary syndromes, gastrointestinal symptoms, acute renal failure, liver dysfunction, hyperglycemia and diabetic ketosis, neurologic deficits, and dermatologic complications. Although these alterations can be due to direct viral infection, indirect mechanisms such as thromboinflammation, dysfunction of the immune system and dysregulation of the renin–angiotensin system have been associated with multiple organ dysfunction (8). All of these are characteristics that resemble the clinical spectrum found on diseases caused by the other aforementioned coronaviruses (9). Additionally, these coronaviruses have similar phylogenetic and clinical characteristics, and different studies have shown that the host immune response can be comparable as well, particularly regarding humoral responses (10–12).

Antibodies against SARS-CoV-2 are essential for outsmarting the virus, as a proper neutralizing response would decrease substantially the number of virions that could successfully infect ACE-2 receptor-expressing cells. Thus, research on antibody responses to SARS-CoV-2 must be a priority for the scientific community responding to the pandemic, both in terms of prophylaxis and treatment.

However, antibody response against this virus is still a subject of controversy and must be addressed carefully. Vaccine effectiveness studies, the possibility of antibody dependent enhancement (ADE) and convalescent plasma therapy, are some of the many topics of debate involving antibody responses to SARS-CoV-2, and plenty of research is yet to be done in some of these fields. However, the robust set of evidence that has surfaced provides clarity in many aspects of the humoral immune response mounted against the novel coronavirus. In this review, we provide an insight on the currently available evidence regarding the nature of antibody response to SARS-CoV-2, especially pertaining to seroprevalence, advances in convalescent plasma therapies, antibody kinetics, and antibody neutralization.

## Methods

**Figure 1:**
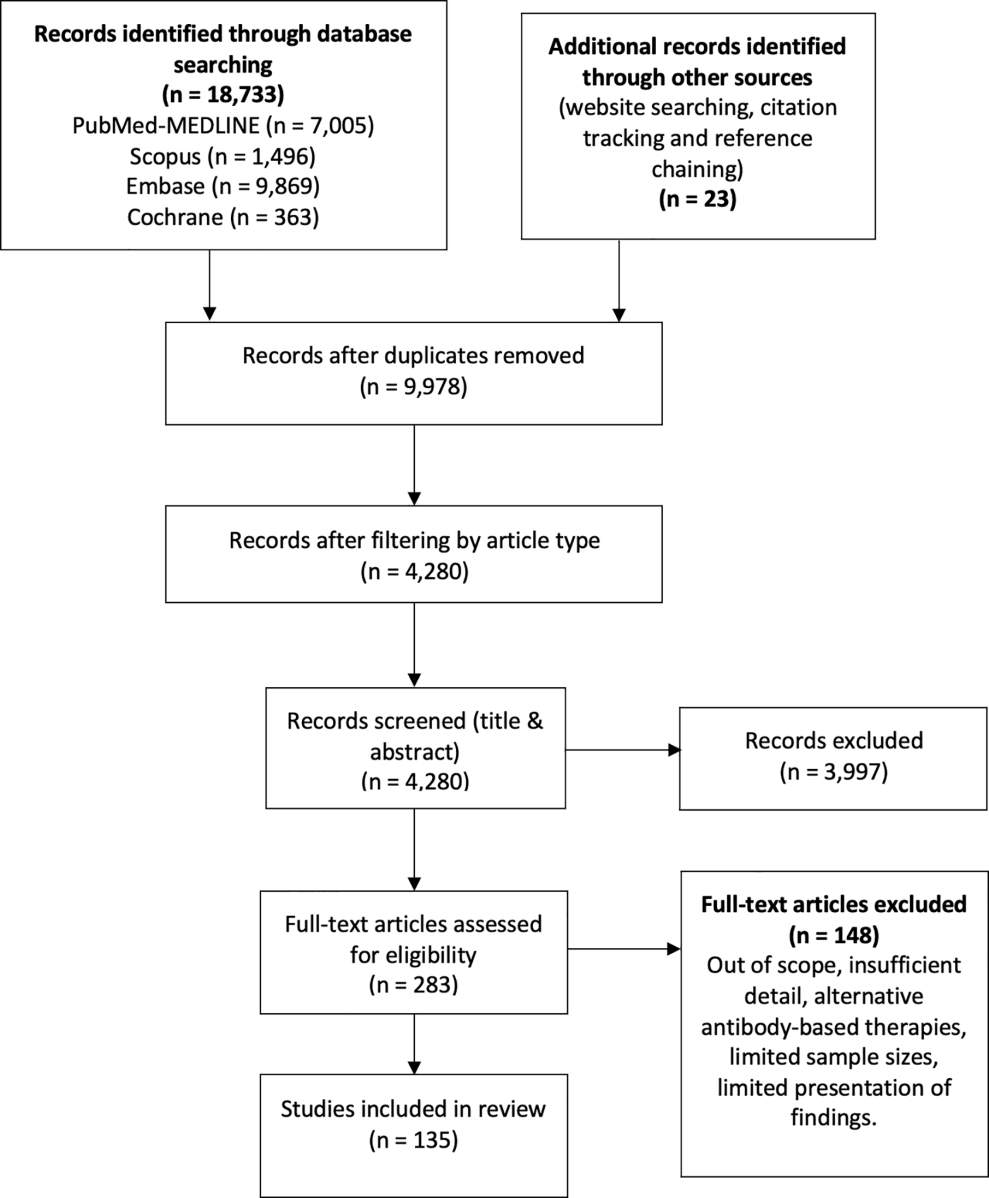
The flow diagram describes the process of literature review on antibody kinetics, neutralization, antibody-dependent enhancement, seroprevalence and convalescent plasma in COVID-19.

We conducted a literature search in the databases PubMed (MEDLINE), Embase, SCOPUS, and Cochrane from inception to 11 March 2021, using the following terms: “SARS-CoV-2”, “COVID-19”, “seroprevalence”, “convalescent plasma”, “neutralizing antibodies”, “antibodies”, “antibody dependent enhancement” and “kinetics”, without geographical restrictions, limited to articles published in English (**Figure 1**). Only articles considered relevant were included according to the authors’ criteria, including original articles, case series, experimental research, reviews, and case reports. The authors’ criteria to consider an article relevant included, among others, the article’s pertinence regarding the specific subjects of seroprevalence, convalescent plasma, kinetics and neutralization, as well as recency and sample size. Technical considerations, opinion articles and performance evaluation articles were not included. Four pre-print articles were carefully revised and included due to their relevance in the fields of study. In addition, the reference lists of each article were reviewed in order to expand the search for relevant articles. Each article underwent a double filter from two authors who schemed the databases according to each topic and deliberated on the relevance of such articles.

### Seroprevalence

The immune response elicited against this coronavirus not only serves the evident physiological purpose of protecting against the infection, but it is also employed for evaluating the impact of this pathogen over a community. Seroprevalence has been important during this pandemic, identifying a significant number of cases that were not diagnosed by the conventional quantitative real-time polymerase chain reaction (qRT-PCR) or by antigen-based tests. As time passes, a growing body of evidence suggests that seroprevalence reflects the dynamics of the pandemic. One such resource is a systematic review and meta-analysis by Bobrovitz et al. that includes 338 studies with over 2.3 million participants (13). Seroprevalence was reported low in general population, and varied among countries and territories. Although this information is of great analytical importance, it must be carefully revised as has not yet undergone peer-review. Another powerful resource is a recent article on The Lancet, which spotlights a dashboard for tracking seroprevalence reports (14). It includes data from 73 countries, the majority of which report seroprevalence below 15%. The data shows variability among territories, with European countries such as United Kingdom, Germany and France reporting higher-range prevalences between 6.7% and 13.6%, in contrast to other countries such as China (0.8-2.46%) or Japan (0.1%). The information displayed can also be analyzed based on local or regional approaches, as nation-wide information is often difficult to gather. Although this information has a worth-mentioning availability and robustness, it is important to analyze individual reports on seroprevalence to characterize further the impact of SARS-CoV-2 over different communities and populations.

The majority of studies on seroprevalence focus on healthcare workers (HCW). Recently, a systematic review and meta-analysis was published by Galanis et al, which showed that the overall seroprevalence was estimated at 8.7% from the information of 49 studies that included 127,480 HCW (15). From this systematic review, it is important to mention that seroprevalence is higher in North American studies while it is lower in Europe, Asia and Africa, but there are also important variations in reports. This can be seen from the data of individual studies. One such example is a report by García-Basteiro et al. in which seroprevalence was reported in HCW from a reference hospital in Spain in June (16). Seropositivity either from IgG, IgM or IgA against SARS-CoV-2 was reported at 9.3%. A German-based study by Brehm et al. showed a notoriously low seroprevalence in HCW from a tertiary care center in November, with an overall seroprevalence of 1.8% (17). Similar studies have been done in China and India, with antibody positivity rates of 17.14% and 11.1% in July and November respectively, in HCW with negative swab samples (18, 19). Data from British centers reported even more dramatic scenarios, with positivity rates of 31.6% from 2,167 HCW in July (20). However, other studies conducted in North American grounds such as that of Stubblefield et al. or that of Hunter et al. display a noticeable heterogeneity, with positivity rates of 7.6% and 1.6% in July and August, respectively (21, 22). A South-American study estimated seroprevalence among HCW at a University Hospital in Colombia in December at nearly 6% (23). These compiled data connote that HCW, who have an augmented exposure to the virus and hence an increased probability of becoming infected, do have an increased seroprevalence as compared to general data from the aforementioned dashboard. Nevertheless, the values are not consistent among them, as they may also be influenced by several factors including social, demographic, professional, and others. In fact, this has been portrayed in individual studies such as that of Goldblatt et al. a cross-sectional study that determined prevalence in eight countries in January (24). Seroprevalence was significantly different in each country, ranging from 0% to 16.93%, and was linked to each national COVID-19 burden. Other individual study by Alseheri et al. showed that this marked variation occurs not only at an international scale (25). This study showed a difference in seropositivity among cities in Saudi Arabia in November, ranging from 0% to 6.31%.

While there is important data on HCW seroprevalence, a significant proportion of the studies have also been carried out in the general population, which reflect more adequately the dynamics and characteristics of the pandemic. An article investigated the seropositivity in May in Wuhan, the city that gave birth to the pandemic, and found a rate ranging between 3.2% and 3.8% (26). There is also data on other countries which have gravely suffered the repercussions of COVID-19, such as Brazil and the United States. Studies in North America by Sood et al., Bryan et al., and Havers et al. show that the prevalence varies according to the specific area, with positivity rates of 4.06%, 1.79%, and up to 6.9% respectively, all between July and August (27–29). In fact, the latter clearly demonstrates the geographical dynamics of the infection, as the authors report that the seroprevalence in the San Francisco Bay area was 1% in contrast to that of New York City, which was 6.9%. Worth noting, Spain, a nation which has suffered greatly from the pandemic as well, conducted one of the greatest epidemiological studies to establish seropositivity. Pollan et al. explored seropositivity through both point of care (POC) -testing and immunoassays in August, with 61,075 and 51,958 tests respectively (30). The seropositivity with the POC tests was 5.0%, while the immunoassays showed 4.6%. Intriguingly, one population-based study found a low seroprevalence in Brazilian territory, a nation that has been hard-struck by the pandemic. This study conducted by Silveira et al. found a seropositivity between 0.048% and 0.222% among three rounds of testing, although it is worth mentioning that this study was based on lateral flow assays (31). These findings are of particular interest because Brazil’s COVID-19 death count has been considerable throughout the pandemic, so it seems that this area of study behaves as an exception, and even in hard-hit countries it is possible to find low-transmission territories. Seroprevalence also shows a more profound impact of COVID-19 in underserved populations. A study in Mumbai from February 2021 showed that seroprevalence in non-slums was 16.1%, while it was as high as 54.1% in slums (32). Furthermore, recent individual studies from late 2020 and early 2021 have shown seroprevalence ranging from 0.09% to 22.2% (33–38). Some of these studies have used a Bayesian approach for accounting for misclassification bias, which represents more properly the data obtained in these seroprevalence studies (35, 36).

**Figure 2:**
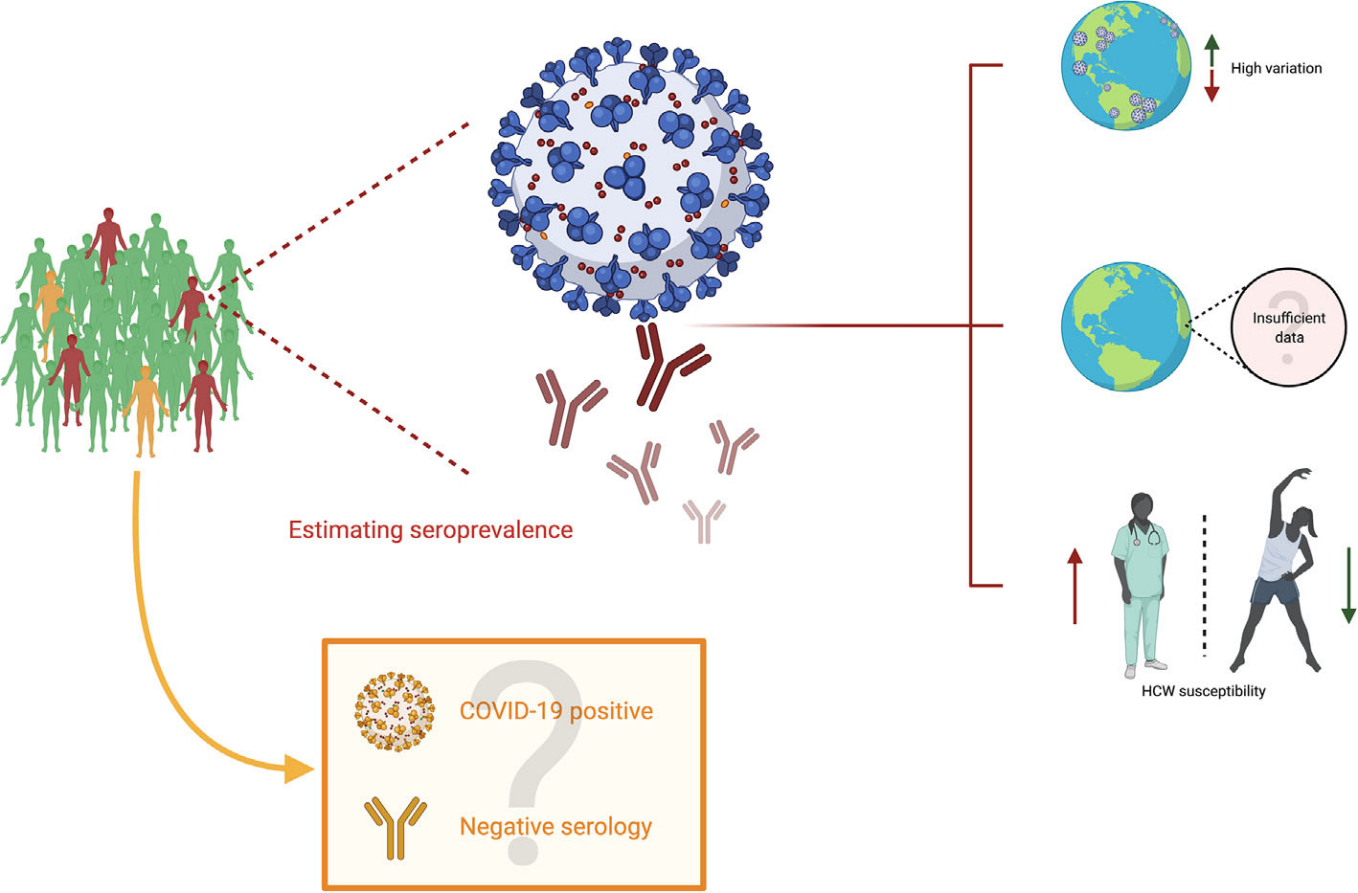
Seroprevalence in COVID-19. Subjects in green are uninfected people (neither current nor past) with negative serology; subjects in orange are infected people (current or past) with negative serology, and subjects in red are infected people (current or past) with positive serology. Estimating seroprevalence points to three primary conclusions. First, there appears to be a high variation among different territories worldwide. Second, although efforts to characterize the impact of SARS-CoV-2 are worth highlighting, there is still insufficient data to estimate the precise impact of this virus. Third, the studies on seroprevalence display the susceptibility of healthcare workers to SARS-CoV-2. However, the impact could be undermined due to individuals who become infected (COVID-19 positive) and have negative serological results.

So far, the available evidence casts a shadow over several points of analysis which are shown schematically in **Figure 2**. First, it leads to the conclusion that seroprevalence is highly variable among territories, and that such values must be read in the light of their particular contexts. Factors such as impact on the community, social and demographic dynamics, economics and political decision-making must be indispensable for an appropriate analysis. Second, the available data on seroprevalence is so far limited, and such challenges must be met by the scientific community so as to describe broadly how this new coronavirus infection affects population on a worldwide scale. Third and last, seropositivity in HCW is evidently greater than that of the general population, which illustrates the risk for medical professionals, highlighting the need for personal protective equipment to be widely available and stresses the need for governments and institutions to strive for the safety of their professionals. For an accurate analysis of these points, it is essential to consider the quality of the antibody tests so as to diminish the incidence of confounding factors, as well as considering the inherent difficulty in estimating the impact when some COVID-19 positive individuals never develop antibodies at all. Finally, time is an important variable to consider because COVID-19 is still spreading with high peaks of infection around the world. Therefore, a dynamic seroprevalence follow-up could be set to have more precise mapping.

### Convalescent Plasma and Therapeutics

**Figure 3:**
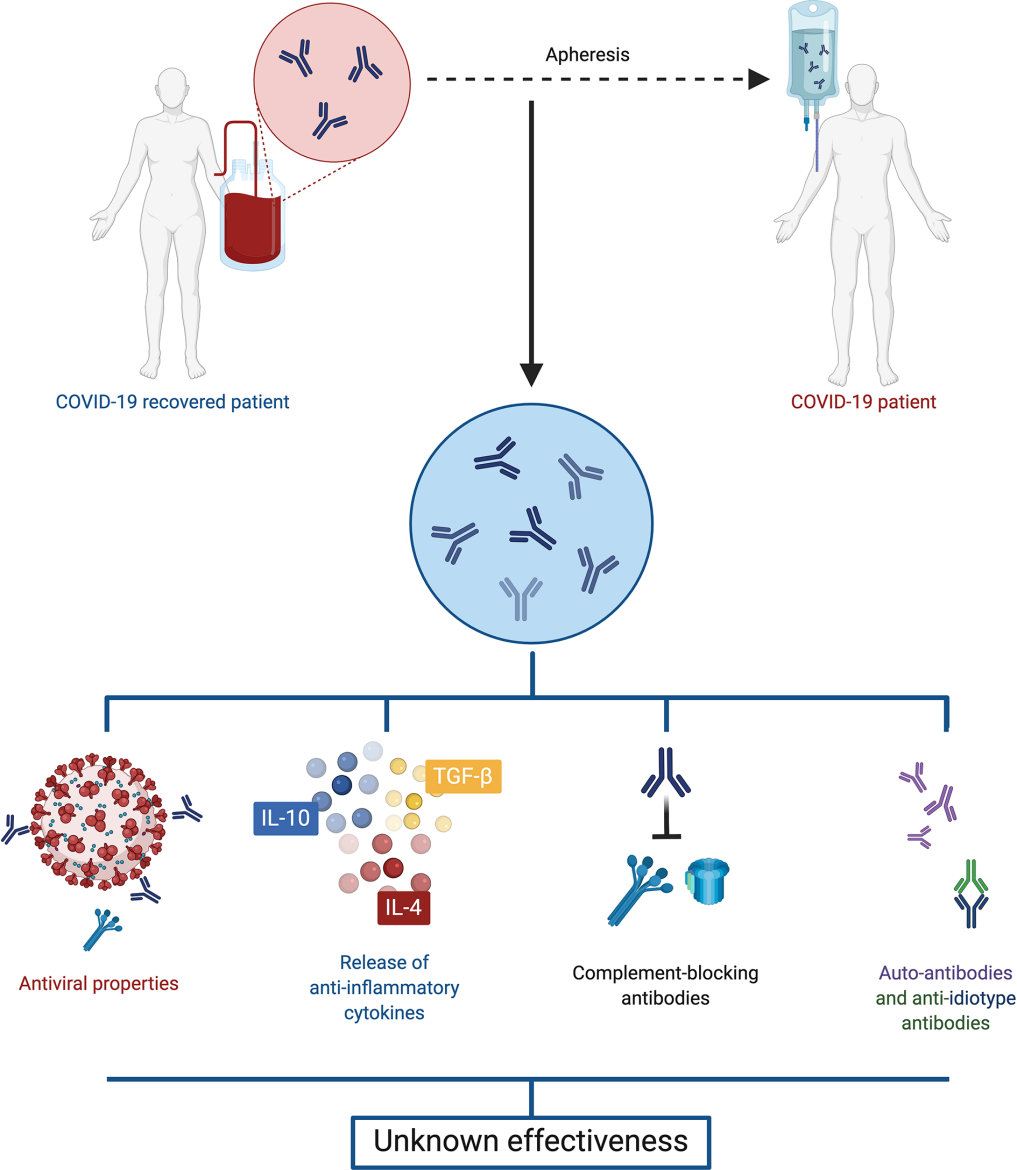
Convalescent plasma. Plasma retrieved from convalescent COVID-19 patients displays both antiviral and immunomodulatory properties, including anti-inflammatory cytokines, complement blocking antibodies, auto-antibodies and anti-idiotype antibodies. However, evidence is so far contradictory, and the effectiveness of this treatment is unclear.

The transfusion of convalescent plasma is a therapy that has been used during the infection of other human coronaviruses, such as SARS-CoV and MERS-CoV (39, 40). Moreover, it has shown to be effective and safe for human use. Convalescent plasma has antiviral characteristics, due to the presence of neutralizing and non-neutralizing antibodies, but it also has immunomodulatory properties through signaling pathways involving anti-inflammatory cytokines, complement blocking antibodies, auto-antibodies, anti-idiotype antibodies and factors involved in hemostasis, depending on the doses used (**Figure 3**) (41).

Recent studies suggest convalescent plasma-treated patients may have increased viral clearance and improvement of clinical manifestations, in particular for fever and radiological findings (42–44), leading to a lower requirement of mechanical ventilation and shorter hospital stay (45). Worth highlighting, a study by Joyner et al. enrolled 21,987 patients who received convalescent plasma (46). Overall, the incidence of all serious adverse events was low, as transfusion reactions, thrombotic events and cardiac events were reported in less than 1% of the population sample. These findings, in accordance with those of other smaller studies, suggest that safety should not be a concern regarding treatment with it (46–49). Further, the time in which it is administered also seems to be decisive in the outcomes of transfused patients, since Joyner et al. showed a 7-day mortality rate of 8.7% (95% CI 8.3-9.2) in transfused patients within 3 days of diagnosis and 11.9% (95% CI 11.4-12.2) in transfused patients after 4 days of diagnosis (50).

However, in terms of grounded, more directly applicable data, a clinical trial could not demonstrate differences in mortality or clinical improvement within 28 days after convalescent plasma therapy (51). It is worth mentioning that this study was limited and did not obtain enough statistical power. Similarly, the PlasmAR trial enrolling 228 patients with severe COVID-19 found no differences between the group of patients treated with convalescent plasma compared to the control group at day 30 (52) and the PLACID trial that included 235 patients with moderate COVID-19, also noted no difference in mortality at 28 days in those who received convalescent plasma compared to those who received only available best standard care therapy (53).

Among the very different reported results, as listed in **Table 1**, uncertainty reigns. A Cochrane systematic review included 19 studies with 38,160 participants and concluded that it is so far unknown whether convalescent plasma is an effective method for reducing severity and mortality in patients with COVID-19 (54). Additionally, reports by regulatory entities have pointed to unimpressive results from these trials (55). Further randomized controlled trials must provide useful information to solve this issue.

**Table 1:** Efficacy and safety of convalescent plasma transfusion as a treatment for COVID-19 patients.

Author	No. of patients	Efficacy	Safety
Abolghasemi	189	No differences in mortality. Lower length of hospitalization and higher discharges.	No adverse events
Cheng	80	Better clinical outcomes with early plasma administration	No adverse reactions
Gharbharan*	86	No improvement in mortality, hospital stay, or day-15 disease severity	No serious adverse events
Joyner	21987	Decrease in mortality rate	Adverse events in less than 1% (transfusion reactions, thrombotic events, cardiac events).
Li*	103	No improvement in clinical within 28 days	2 patients had an adverse event after transfusion that improved with supportive therapy
Xia	138	Symptoms improvement and lower mortality rate	No serious adverse events

*Randomized clinical trials. Dpt, Days post-transfusion.

Measuring antibody titers before transfusions is required in most cases, considering that the antibody responses in recovered patients is extremely variable. Studies have reported that around 33% of patients had titers less than 1:50 (56). Besides, it appears that mild patients may have lower neutralizing antibody titers compared to severe patients (57). In fact, only hospitalized patients who were not receiving mechanical ventilation and received plasma transfusions with higher antibody titers appear to have benefit in clinical outcomes (50). Also, in an animal model with green monkeys, it was noted that those who received higher antibody titers had lower SARS-CoV-2 levels in the respiratory compartments, less severity of virus-related lung pathology, reduction in coagulopathy and inflammatory processes (58).

Thus, considering that neutralizing antibodies could be presumed to be potentially protective during SARS-CoV-2 infection, it is necessary to know the status of neutralizing antibodies in donor plasma. However, due to the difficulty in performing neutralization assays, the nucleoprotein (NP), spike (S) 1 and S2 specific IgG could be an alternative for the indirect measurement of neutralizing antibodies in donor plasma (57). Notably, the antibody response against receptor-binding domain (RBD) has been recognized as anti-SARS-CoV-2 effective in donor plasma (56); and expansion of clones of RBD-specific memory B cells was higher in recovered patients (59). The application of these data, which resulted from more specifically focused studies and pertains to aspects of basic research, must be warranted and taken into account for designing clinical studies in a stride to solve the uncertainty mentioned before.

### Kinetics

**Figure 4:**
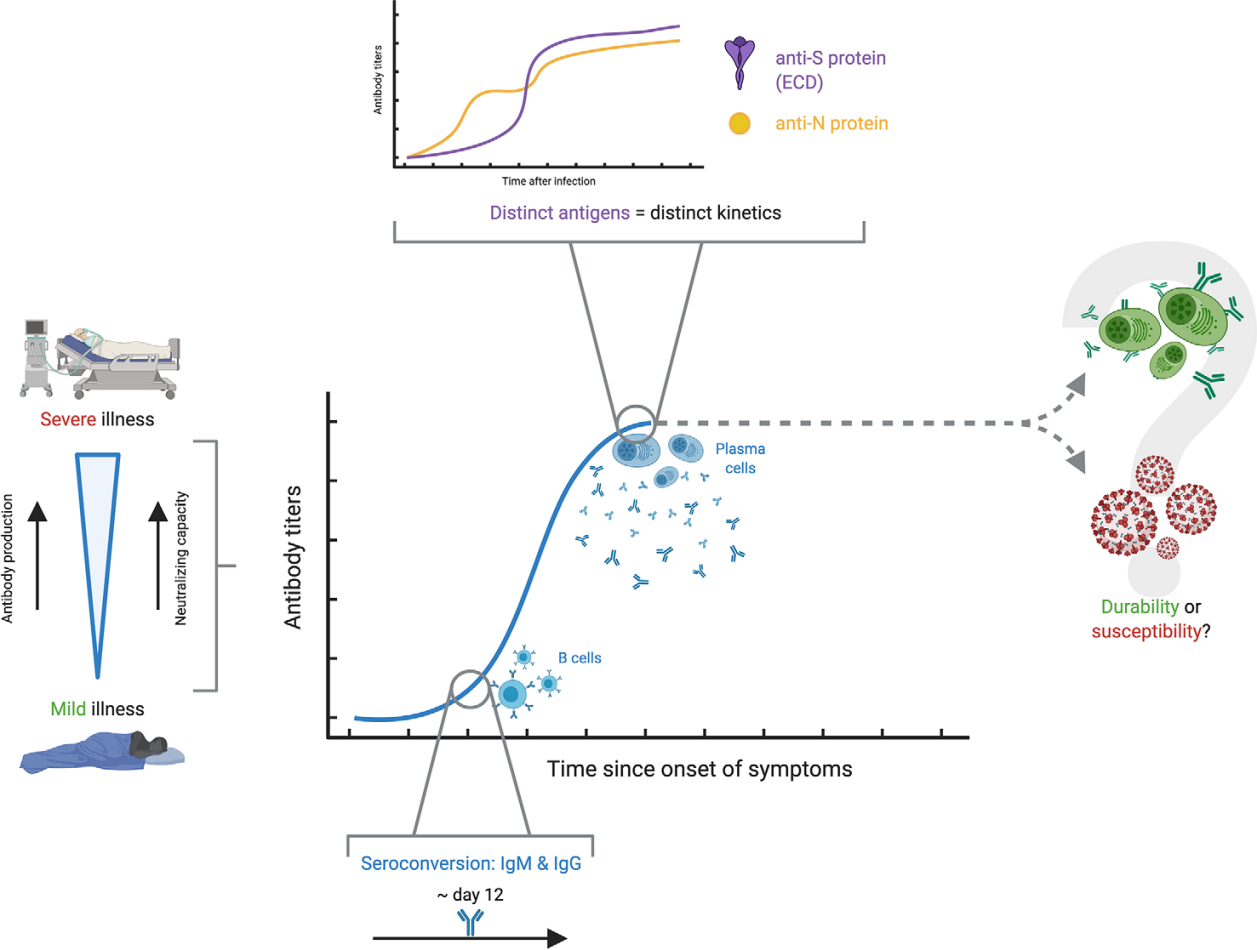
Antibody kinetics in COVID-19. The antibody kinetics show to be highly variable among individuals, but there seems to be a clear correspondence between severe disease, high antibody production and high neutralizing capacity, while the opposite is true with mild disease. Among the different studies, seroconversion appears at around 12 days. A characteristic finding is that there appears to be distinct kinetic profiles for the different antigens against which antibody responses are developed. The most important question which remains unsolved is whether antibody titers persist over time conferring protection, or if, on the contrary, antibody production wanes over time and renders people susceptible to reinfection.

A fundamental, yet roughly understood matter in the humoral response to SARS-CoV-2 is the kinetics, whose importance is stressed by the need for a robust and long-lasting immunity against this coronavirus. Despite the narrow understanding on antibody kinetics in the context of COVID-19, four areas of knowledge have seen noticeable advances: differences in antibody responses between critical and non-critical COVID-19 patients; seroconversion and magnitude of antibody responses during the first weeks after infection; variability in antibody kinetics depending on their respective antigen targets; and controversy in the reliability and neutralization capacity of antibodies through time (**Figure 4**). It is worth mentioning that kinetics, performance, analytical and clinical validation of specific assays are also related to the topic of antibody kinetics in COVID-19, and have been broadly explored elsewhere (60–64).

Perhaps one of the clearest and most well-understood topics regarding antibody kinetics is the relationship with severity. The available evidence strongly suggests that severity is associated with higher levels of antibodies. Rijkers et al. showed results in a sample with 38 severe and 24 mild patients, in which the severe group developed a robust antibody response with adequate neutralizing capacity, in contrast to a mild group with only 75% seropositivity and poor neutralizing capacity (65). Several reports, including systematic reviews, opinion articles and original articles, point towards this same conclusion, strongly asserting that severity in COVID-19 patients seems to be associated with antibody production and response against this virus, although the immunological, virological or physiological bases behind this are so far unknown (66–69). Still, further research must be done on the topic, as the findings in some studies suggest differently. For example, a study by Wang et al. with 12 severe and 11 mild COVID-19 patients showed that, even though mild patients had significantly lower IgM titers compared to their severe counterparts, both groups showed comparable IgG responses at 9 days post-onset of symptoms (POS) (57). Recent evidence suggests that there is a prognostic relationship between antibody kinetics and severity of the disease in COVID-19. A report by Ren et al. has shown the correlation in severe COVID-19 patients with a delayed antibody response, in contrast to an earlier-mounted response by mild patients (70). Although the study has a limited follow-up, further data presented by Lucas et al. in a recent preprint leads to similar conclusions. These researchers showcased that not only is there a delayed antibody response in lethal COVID-19, but also that an early-mounted neutralizing response correlates with discharge (71). The authors speculate on a possible window of 14 days POS in which antibody responses must be mounted for increasing odds of survival. Altogether, these results pave the way towards an understanding of antibody kinetics and how it relates, and even dictates, COVID-19 severity.

The kinetics of seroconversion has also been evaluated in several studies. Large studies have shown seroconversion rates from 91 to 99% (72, 73). Lynch et al. point out that over 80% of patients had IgM and IgG seroconversion between 8- and 10-days POS, and highlight that patients admitted to the intensive care unit had higher peak measurements in all intervals between 6 and 20 days for IgM (74). Additionally, a different study by Orth-Höller et al. shows that positive IgG titers were found in most mild and moderate patients after two to three weeks (75). In contrast, Zhang et al. described that the production of IgM and IgG was delayed in the critical group and reached the peak at 1-month POS (76). Several studies, including an aforementioned meta-analysis, suggest that seroconversion of both IgM and IgG occurs at around 12 days POS with extensive variation, but does not shed any light towards severity (66), underlying the importance of further studies (66, 74–78). This evidence on the first phase of antibody response in COVID-19 resembles that for other coronaviruses. In other outbreak-related coronaviruses such as SARS-CoV, as well as endemic coronaviruses such as HCoV-229E, a noticeable increase in antibody response was detected 10-20 days after the onset of illness (67, 79–81).

Naturally, antibody profiles and dynamics vary depending on the isotype of interest (70, 82), but evidence has also gathered to indicate that there are dissimilar profiles of antibody kinetics depending on the antigen of interest, and consequently natural antibody levels vary significantly and cannot be characterized as a whole (83). An article by Chen et al. portrays this avidly, as serum IgM and IgG responses displayed distinct kinetic profiles against NP, RBD, S1 and the ectodomain (ECD) of the S protein (70, 84). This heterogeneity may be used in favor of the diagnostic precision, as combined detection of antigens such as NP and ECD increases test sensitivity, as well as a combination of IgM or IgG specific against N or S (84, 85). Nonetheless, attention should also be set on other, less popular antigens. There is evidence proving that targets such as ORF8 and ORF3b elicit noticeably strong antibody responses and provide very high specificity and sensitivity for evaluating antibody response, even outperforming serological assays screening for other antigens such as S or N protein (86). All of these findings further demonstrate that evidence on antibody response in terms of kinetics is worth investigating, as it could provide insight on COVID-19 diagnosis and the maintenance of durable immunity.

The main concern on antibody response kinetics to SARS-CoV-2 is the establishment of a durable protection. Based on the available evidence, it is yet to be determined if the antibody response effectively wanes over time, and if the resulting antibody titers are enough to provide protection against reinfection (87). In fact, several reports show an evident decrease in antibody response over time, which depict variable declining neutralizing antibody titers during the follow-up period of convalescent patients (68, 88). Still, it is unclear whether the quality of the remaining antibodies is enough to effectively neutralize the virus (69). These concerns are reinforced by the recently published reports on confirmed reinfections, although more research must be conducted in order to determine the nature and causes of such phenomena, and what is the role of antibody kinetics, if any (89, 90). However, there are also several reports that point to the opposite conclusion. There is compelling evidence on the persistence of robust neutralizing antibodies for months after infection, as well as favorable antibody kinetic profiles in large national studies, which range from five to eight months (72, 73, 91, 92). To assess said establishment of a durable protection, Lumley et al. studied antibody responses in healthcare workers and followed up 1265 seropositive subjects (93). Among these participants, who were followed up to 31 weeks, 2 had a positive PCR test. Other even larger studies have pointed to similar conclusions, as Abu-Raddad et al. followed 43,000 PCR-positive subjects and found an estimated incidence rate of reinfection of 0.66 per 10,000 people-weeks (94). Although this conclusion does not involve humoral response directly, it does indicate that immune protection is robust among those previously infected and that the risk of reinfection is low, and that it is mostly asymptomatic.

A parallel can be drawn over kinetics with that of other coronaviruses, as antibody responses to MERS-CoV and SARS-CoV effectively wane over time. Indeed, antibody responses can be detected in MERS patients one year after the infection, but in a notably lower concentration (95). A similar phenomenon occurs in SARS patients, as some studies have shown that antibodies can be found 2-3 years after the infection, albeit in low titers (67, 81, 96). Although data cannot be directly extrapolated from one virus to another, it is safe to assume that the data presented herein suggests the kinetics of antibodies against SARS-CoV-2 might follow a similar pattern to those of other outbreak-related coronaviruses, as antibody responses are detected in patients previously infected with SARS-CoV-2, but appear to wane as well.

### Neutralizing Antibody Response: Importance and Considerations on Its Effect

Neutralizing antibodies (NAbs) are often correlated with long-term immunity in several viral infections (97). Hence, understanding the presence of NAbs in SARS-CoV-2 infection might be helpful as well. Most infected patients with SARS-CoV-2 develop variable titers of NAbs between days 14 and 20 POS (98). A better perspective on NAb production might be achieved upon a well-established comparison between age groups. In children, the onset and synthesis of NAbs seems to be similar than in adults, so most of them produce titers of neutralizing antibodies of variable level. Of note, Pengcheng Liu et al. suggest that NAbs produced during the acute phase may be insufficient for viral clearance, which could be associated with prolonged viral shedding (99).

Another important aspect surrounding antibody neutralization against SARS-CoV-2 is the influence of immunity against seasonal human coronaviruses (HCoV), and whether or not such immunity protects against SARS-CoV-2. Some published results so far suggest that there is probably no cross-neutralizing response between SARS-CoV and SARS-CoV-2 (100), which renders unlikely that previous infections with SARS-CoV provide protection against infection by SARS-CoV-2. However, other reports show the opposite. Some authors have demonstrated a partial protection for SARS-CoV-2 in patients who previously were infected by SARS-CoV (101). These studies highlight a potential of cross-reactivity from seasonal and endemic coronaviruses with SARS-CoV-2 (102). In fact, not only does it appear to exist a comparable neutralization activity between people infected by SARS-CoV-2 and those infected by different HCoVs, but evidence has surfaced as well on the interference upon entry of SARS-CoV-2 into target cells in those patients who had previous exposure to seasonal coronaviruses (103).

**Figure 5:**
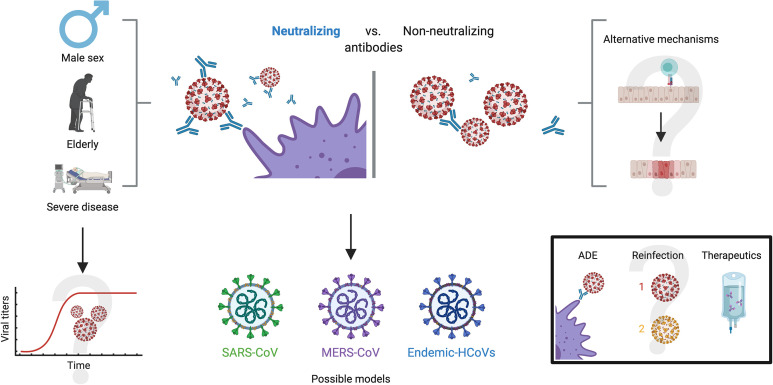
Antibody neutralization in COVID-19. Three characteristics appear to hold a close relationship with high antibody neutralization capacity: male sex, old age and severe disease. A possible explanation is viral persistence in patients with these characteristics, but this is yet to be confirmed. On the other side of the spectrum, alternate mechanisms have been proposed for patients that do not develop robust neutralizing antibodies, such as high cytotoxic activity and a robust innate immune response, although these remain undetermined. For investigating neutralizing dynamics against SARS-CoV-2, other models with similar coronaviruses have been explored, such as SARS-CoV, MERS-CoV and endemic CoVs. Further investigation on neutralizing capacity will present definitive solutions to the enigmas of antibody-dependent enhancement, likelihood of reinfection and the effectiveness of treatments such as convalescent plasma.

Besides, it has been proven that neutralizing antibodies have a positive correlation with age, male sex and severity of the disease (**Figure 5**) (104). Wu et al. reported that NAbs were higher among older patients compared to those middle-aged and younger, being the latter those who had lower neutralizing titers (105). Moreover, they found that titers of NAbs were positively correlated with the C-reactive protein levels and negatively with lymphocyte count at the time of admission. Of note, younger people do not necessarily develop low titers, as the same study also detected 2 patients with very high neutralizing titers (ID50:15989, 21567) in this age group, prompting other factors to be considered.

Regarding differences by gender, and despite the fact that men and women seem to have the same risk of infection by this virus (106), there are significant differences in the course and outcome of the disease between them, and worse outcomes and higher NAbs titers among the male population (98, 104, 107). Indeed, clinical outcomes show that males experience both a higher severity and fatality for COVID-19 infection than females (108, 109). Worth noting, an article by Takahashi et al. highlights the importance of specific cytokines such as IL-8 and IL-18, as well as the role of non-classical monocytes and the significantly diminished T cell responses in male patients with unfavorable outcomes (110). These findings demonstrate, altogether, that there are profound physiological differences between males and females in terms of COVID-19 progression and immunity to the virus.

Similar to the wide humoral response, the severity of COVID-19 has been correlated with titers of neutralizing antibodies against SARS-CoV-2, as these are higher among hospitalized patients or those in intensive care units (ICU) compared to patients with mild symptoms (111, 112). Although some neutralizing antibodies with strong RBD region binding affinity have been found in convalescent patients, (113), currently hospitalized patients have neutralizing titers up to 3000-fold higher than outpatients and donors of convalescent plasma (114). Despite the fact that most of the mechanisms behind these phenomena are unknown, there are several hypotheses. Some authors discuss the possibility that high titers are found in severe patients as a consequence of the strengthened and prolonged B-cell receptor stimulation (115), which might be associated with insufficient viral clearance. They also shed light on the potential role of other immune mechanisms in those with less severe outcomes. One such example might be a robust CD8+ T cell response that could confer protection against the virus more effectively, but this is yet to be proven by future investigations (115). Correlations between neutralizing capacity and disease severity have also been drawn in the context of other coronaviruses such as MERS-CoV, where peak overall antibody and neutralizing antibody levels increase in cases of severe disease, while mild and asymptomatic cases appear to have little or no neutralizing antibodies (116, 117).

The overall timing and kinetics of NAbs is a topic worthy of discussion. As mentioned in “Kinetics”, NAbs are detected earlier in non-lethal and less severe patients, and a timeframe of 14 days has been proposed to classify patients at risk of having lethal outcomes (70, 71). In fact, this might suggest a contrary immune phenotype to that of quick COVID-19 healers suggested by Chen et al. (118). These authors showed that there is a specific group of mild patients who have a shortened disease course and a sustained antibody production for nearly 100 days. Ren et al. also showed a general correspondence between the timing of NAbs detection and anti-RBD and anti-S antibody detection, thus showing that NAbs appear in a similar time frame to these other antibodies (70). The duration of NAbs is also subject to intensive research since it could provide information about protective immunity over time. However, the duration of circulating NAbs in convalescent people has not been established yet. Then, it can only be speculated based on the information on other coronaviruses, such as SARS-CoV, which has neutralizing antibodies for up to 24 months post-infection (49). Other than that, information could be extrapolated from general kinetic profiles reported by other studies, as shown above in this review.

Yet another topic which is subject of public debate regarding neutralizing antibodies is the relationship with antibody dependent enhancement (ADE) and reinfection. ADE is a phenomenon in which, contrary to the ideal condition of neutralizing antibodies, the immune system produces suboptimal antibodies that are not able to inhibit the replication cycle and, instead, facilitate viral entry to susceptible cell types (119–121) Since the beginning of the pandemic, ADE has been a possibility noted by many authors due to past experiences with other coronaviruses (122), although concrete evidence has been limited (123–127). Some authors argue that the possibility of ADE would have therapeutic implications that should be addressed urgently, in particular with vaccines, hyperimmune globulin and convalescent plasma (128, 129). Others have speculated on the possible relationship between ADE and severity in COVID-19 patients (122, 130), while some have even suggested that ADE is responsible for specific conditions such as multisystem inflammatory syndrome in children or hyperinflammation in people living with COVID-19 (131, 132). In addition, the potential role of ADE has been suggested in the Nevada reinfection case, but aside from speculation, there is no evidence or support for this hypothesis (90).

However, there is also an important number of authors who have speculated from other perspectives on ADE in COVID-19. For instance, some authors have suggested that convalescent plasma safety studies weigh against the possibility of this phenomenon in SARS-CoV-2 infection, lessening the concerns, although they do not discard this possibility (133, 134). Altogether, the multiple articles garnered about ADE display a wide spectrum of opinions on a subject that is yet to be deeply investigated and must remain within sight (**Figure 5**). Nevertheless, it is necessary to state that the available evidence so far on ADE and its potential role on infection by SARS-CoV2 suggests that this phenomenon is unlikely and would have limited inherence in the clinical course of COVID-19, but compelling evidence is yet to be presented on the subject, either for or against.

## Discussion

The nature and importance of antibodies against SARS-CoV-2 has been a subject of debate. Despite the ample areas of research on humoral response in COVID-19, neutralization and antibody kinetics have been at the center of investigation in humoral immune responses against this coronavirus. The disparities among people in these two subjects appears to be finely tuned and hold an important correspondence with the severity of the disease. As shown in this review, the antibody response, its duration and its capacity to confer protection, appears to behave more favorably in patients who had coursed with severe disease as compared to those with milder symptoms.

There is compelling evidence, indicating that some immune functions are misregulated in COVID-19 patients, such as loss of germinal centers with poor Bcl-6 activity, leading to an overall inadequate immune response (135), that may account for the few confirmed cases of reinfection (136) and, perhaps, for the persistent circulation for SARS-CoV-2. Additionally, considering the apparently low risk of ADE in the context of COVID-19 with the evidence garnered so far, the humoral immune response does not likely represent a danger in itself.

Investigating and reviewing seroprevalence in COVID-19 gives perspective on the impact of this virus over time. So far, few major studies have led conclusively to seropositivity rates that can be easily extrapolated to the general population, and that could assist analyzing the “protected population” status (14, 15). Therefore, COVID-19 response urgently needs to be enlightened with more representative data, in particular defining the neutralizing capacity of the humoral response and its duration. This information would shed light on the truly protected population compared to those which might be susceptible to infection, even if they have encountered this virus before.

Convalescent plasma is another subject that provides perspective on antibodies. Several clinical trials are currently investigating the efficacy of such a treatment at different stages of the disease, compared to different treatments and interventions (137–142). Current information has also been contradictory, as some have reported beneficial effects of convalescent plasma while others have criticized the lack of controlled groups in the studies published so far, as well as randomized clinical trials suggesting that the balance of efficacy and adverse events is still uncertain (51, 143).

Both SARS-CoV-2 specific CD4 + and CD8 + T cells, as well as B cells against SARS-CoV-2 epitopes, have been found for up to 6 months after infection in about 95% of COVID-19 patients (91). The same response that occurs with natural infection could be expected with vaccines, such as those developed by Pfizer-BioNTech, Moderna and Oxford-Astrazeneca, since they stimulate the humoral immune response directed to the SARS-CoV-2 spike protein (144–146) At the same time, it has also been seen that although the memory immune response remains for several months, it decreases over time; such is the case of the neutralizing antibody response and spike-specific CD4 + T cells, which diminish in the former 4 months post-infection. However, it seems that S-specific IgG+ memory B cells accumulate over time (147) and may be responsible for maintaining the efficacy of vaccines at long term. It should be noted that the possibility that the SARS-CoV-2 vaccines are only transiently protective in the population cannot be ruled out, which leads to the belief that the vaccines may require greater immunogenicity than natural infection or revaccinate the general population periodically, in order to maintain long-time protection.

Altogether, the evidence on antibody responses to SARS-CoV-2 is broad and ever-growing, but there are still important contradictions and uncertainties that must lead the scientific community to find better evidence that can provide a more precise scope for understanding the biological phenomena, and at the same time lead clinicians to find the best possible interventions for their patients.

## Author Contributions

MC-M and YM-C reviewed the literature and prepared drafts of the manuscript for final submission. PP, PV, and MR assisted in evaluation of the literature and revised the drafts of the manuscript. All authors contributed to the article and approved the submitted version.

## Funding

Project BPIN 2020000100131 and Universidad de Antioquia.

## Conflict of Interest

The authors declare that the research was conducted in the absence of any commercial or financial relationships that could be construed as a potential conflict of interest.
